# Chloroplast genome assemblies and comparative analyses of commercially important *Vaccinium* berry crops

**DOI:** 10.1038/s41598-022-25434-5

**Published:** 2022-12-14

**Authors:** Annette M. Fahrenkrog, Gabriel O. Matsumoto, Katalin Toth, Soile Jokipii-Lukkari, Heikki M. Salo, Hely Häggman, Juliana Benevenuto, Patricio R. Munoz

**Affiliations:** 1 Horticultural Sciences Department, University of Florida, Gainesville, FL 32611 USA; 2 Ecology and Genetics Research Unit, University of Oulu, 90014 Oulu, Finland; 3 Inari Agriculture Nv, Industriepark Zwijnaarde 7a, 9052 Ghent, Belgium

**Keywords:** Genetic markers, Comparative genomics, Plant breeding, DNA sequencing, Phylogenetics, Plant domestication

## Abstract

*Vaccinium* is a large genus of shrubs that includes a handful of economically important berry crops. Given the numerous hybridizations and polyploidization events, the taxonomy of this genus has remained the subject of long debate. In addition, berries and berry-based products are liable to adulteration, either fraudulent or unintentional due to misidentification of species. The availability of more genomic information could help achieve higher phylogenetic resolution for the genus, provide molecular markers for berry crops identification, and a framework for efficient genetic engineering of chloroplasts. Therefore, in this study we assembled five *Vaccinium* chloroplast sequences representing the economically relevant berry types: northern highbush blueberry (*V. corymbosum*), southern highbush blueberry (*V. corymbosum* hybrids), rabbiteye blueberry (*V. virgatum*), lowbush blueberry (*V. angustifolium*), and bilberry (*V. myrtillus*). Comparative analyses showed that the *Vaccinium* chloroplast genomes exhibited an overall highly conserved synteny and sequence identity among them. Polymorphic regions included the expansion/contraction of inverted repeats, gene copy number variation, simple sequence repeats, indels, and single nucleotide polymorphisms. Based on their in silico discrimination power, we suggested variants that could be developed into molecular markers for berry crops identification. Phylogenetic analysis revealed multiple origins of highbush blueberry plastomes, likely due to the hybridization events that occurred during northern and southern highbush blueberry domestication.

## Introduction

The genus *Vaccinium* L. (family Ericaceae) comprises more than 450 species of wide geographic distribution, occurring mostly in the Northern Hemisphere and in mountainous regions of tropical Asia, Central and South America. With a few exceptions, most of the berry fruits produced by the genus are edible by both birds and mammals^1^. Some species have become economically important crops over the past century, being either bred and cultivated in commercial fields, or harvested from managed wild stands^2^. The major commercial crops are northern highbush blueberries (*V. corymbosum* L.), southern highbush blueberries (*V. corymbosum* L. hybrids), lowbush blueberries (*V. angustifolium* Aiton), rabbiteye blueberries (*V. virgatum* Aiton), bilberries (*V. myrtillus* L.), cranberries (*V. macrocarpon* Aiton), and lingonberries (*V. vitis-idaea* L.). In addition to their pleasant flavors, the nutritional value of these berries has led to a significant increase in consumption and production worldwide. In the United States alone, the wholesale value of the *Vaccinium* berry industry exceeds US$1 billion per year^3^.

Given the diversity and complexity of the genus *Vaccinium*, it has been further divided into more than 33 sections or subgenera^4^. The most important *Vaccinium* crop species are found in the sections *Cyanococcus* (blueberries), *Oxycoccus* (cranberry), *Vitis-Idaea* (lingonberry), and *Myrtillus* (bilberry)^5^. However, species and section delimitations have been extensively discussed in the literature, as they do not form monophyletic groups^6,7^. The taxonomic classification has been difficult to resolve because of considerable phenotypic variability with overlapping morphologies, complex ploidy series (ranging from diploids to hexaploids), and general lack of crossing barriers leading to numerous hybridization events^5^. As a result, some species are burdened with an extensive synonymy according to different authors^1,8,9^. Nevertheless, this great diversity and intra-/inter-sectional cross-compatibility have been exploited by breeding programs, allowing for the introduction of useful traits from many species^10–14^. Interspecific hybridizations within the *Vaccinium* section *Cyanococcus*, for example, have played a critical role in the development of low chill southern highbush blueberries through numerous crosses of northern highbush blueberry with warm-adapted Florida native species^10,15^.

A few studies have used molecular data to perform phylogenetic analyses of the genus and relevant sections, including the use of simple sequence repeats^16^, chloroplast *matK* and *ndhF* genes and the nuclear ribosomal ITS region^17,18^. These studies have supported the polyphyletic status of current taxonomic groups and were not able to resolve close relationships. With the decreasing costs of next-generation sequencing, using the whole plastome as a “super-barcode” is becoming a popular strategy for increased resolution at lower plant taxonomic levels^19–21^. Moreover, the genetic properties of chloroplasts (i.e., uniparental inheritance, haploid, and non-recombinant nature) can simplify phylogenetic reconstructions when dealing with mixed-ploidy species, and facilitate the usage of their polymorphic sites as molecular markers. However, only a few *Vaccinium* chloroplast genomes have been published so far, with most of these studies reporting only the plastome assembly, without performing comparative analyses^22–29^. Moreover, organellar genomes of horticultural plants are overall underrepresented in databases^30^.

Chloroplast-based molecular markers can be particularly useful for fast berry product authentication. Berry crops represent a set of high-value healthy fruit species, and adulteration commonly occurs by the fraudulent replacement of high-value berries with lower value counterparts (e.g., wild bilberries with cultivated blueberries) or by mistakenly identifying *Vaccinium* berries during labelling^31,32^. In addition, chloroplast genome sequences are important for breeding and biotechnology purposes given that species-specific sequences facilitate codon optimization and provide best regulatory sequences for genetic engineering of chloroplasts that could enhance translation and transgene integration^33^.

By generating additional chloroplast genome sequences for economically relevant *Vaccinium* species, we aim to provide valuable resources to assist future taxonomic and domestication studies, the development of molecular markers for berry crops identification, and a framework for chloroplast biotechnology. Therefore, in this study, we report the assembly of five new *Vaccinium* chloroplast sequences representing the following economically relevant berry types: northern highbush blueberry—NHB (*V. corymbosum*), southern highbush blueberry—SHB (*V. corymbosum* hybrids), rabbiteye blueberry (*V. virgatum*), lowbush blueberry (*V. angustifolium*), and bilberry (*V. myrtillus*). We compared the assemblies in terms of synteny and gene content and identified polymorphic sites that could be developed into molecular markers for berry food product identification since we included representative samples of major economically important *Vaccinium* berry types in the analyses. We also performed whole plastome phylogenetic analyses including other available *Vaccinium* sequences.

## Results

### Chloroplast genome assembly

The whole genome sequencing reads used to assemble the chloroplast DNA (cpDNA) of the five *Vaccinium* species were obtained using two different sequencing platforms: (i) a PacBio long reads approach was used to sequence SHB and rabbiteye, and (ii) an Illumina short reads approach was used for NHB^34^, lowbush, and bilberry.

Complete cpDNA assemblies (sequences without any gaps) were obtained for SHB and rabbiteye using PacBio long reads. A total of 20 contigs (longest contig: 277,507 bp) were assembled for SHB, while the rabbiteye assembly generated two contigs (longest contig: 233,010 bp). Given the length of the complete cpDNA from a related species (cranberry) downloaded from GenBank (~ 176 kb)^23^, the longest contigs of SHB and rabbiteye were likely to contain the complete cpDNA sequence. When the longest contigs were circularized, redundant sequences from their termini were trimmed. The assemblies were further polished, yielding the final SHB and rabbiteye assemblies of length 191,378 and 195,878 bp, respectively (Table 1).

**Table 1:** Assembly and annotation statistics of the chloroplast genomes from six *Vaccinium* species.

*cpDNA*	SHB	Rabbiteye	NHB	Lowbush	Bilberry	Cranberry
Species	*V. corymbosum* hybrids	*V. virgatum*	*V. corymbosum*	*V. angustifolium*	*V. myrtillus*	*V. macrocarpon*
Genotype	Arcadia	Ochlockonee	Draper	Brunswick	OU-L2	Stevens
Sequencing	PacBio/Illumina	PacBio/Illumina	Illumina	Illumina	Illumina	PacBio
Assembler	Canu	Canu	Novoplasty	Novoplasty	Spades/CAP3	Canu
Genome size (bp)	191,378	195,878	186,057	182,334	191,744	176,095
Number of gaps (stretch of Ns)	0	0	2	5	16	0
LSC size (bp)	106,385	106,427	105,714	107,607	107,134	104,591
SSC size (bp)	3037	3035	3027	2997	3008	3028
IR size (bp)	40,978	43,208	38,658	35,865	40,801	34,238
Total number of genes*	112 (136)	112 (136)	112 (136)	112 (139)	112 (145)	112 (134)
Protein-coding genes*	74 (85)	74 (85)	74 (85)	74 (85)	74 (89)	74 (85)
tRNA genes*	34 (43)	34 (43)	34 (43)	34 (46)	34 (48)	34 (41)
rRNA genes*	4 (8)	4 (8)	4 (8)	4 (8)	4 (8)	4 (8)
GC%	36.8	36.8	36.8	36.8	36.6	36.8
Accession Number	OM791342	OM791343	BK061167	OM791344	OM809159	MK715447.1
Reference	This work	This work	This work	This work	This work	Diaz-Garcia et al. 2019

*Number of unique functional genes. In parentheses: Number of genes including duplicates.

The NHB, lowbush and bilberry cpDNAs were obtained from short reads only, resulting in lower quality assemblies compared to the SHB and rabbiteye cpDNA sequences. The short-read assemblies yielded several contigs and reference-guided scaffolding was performed to obtain a single pseudomolecule. The polishing procedure and the placement of a consensus inverted repeat into the sequences were collectively able to close some gaps, although a few remained. The final draft cpDNA assemblies had 186,057, 182,334, and 191,744 bp for NHB, lowbush and bilberry, respectively (Table 1).

**Figure 1:**
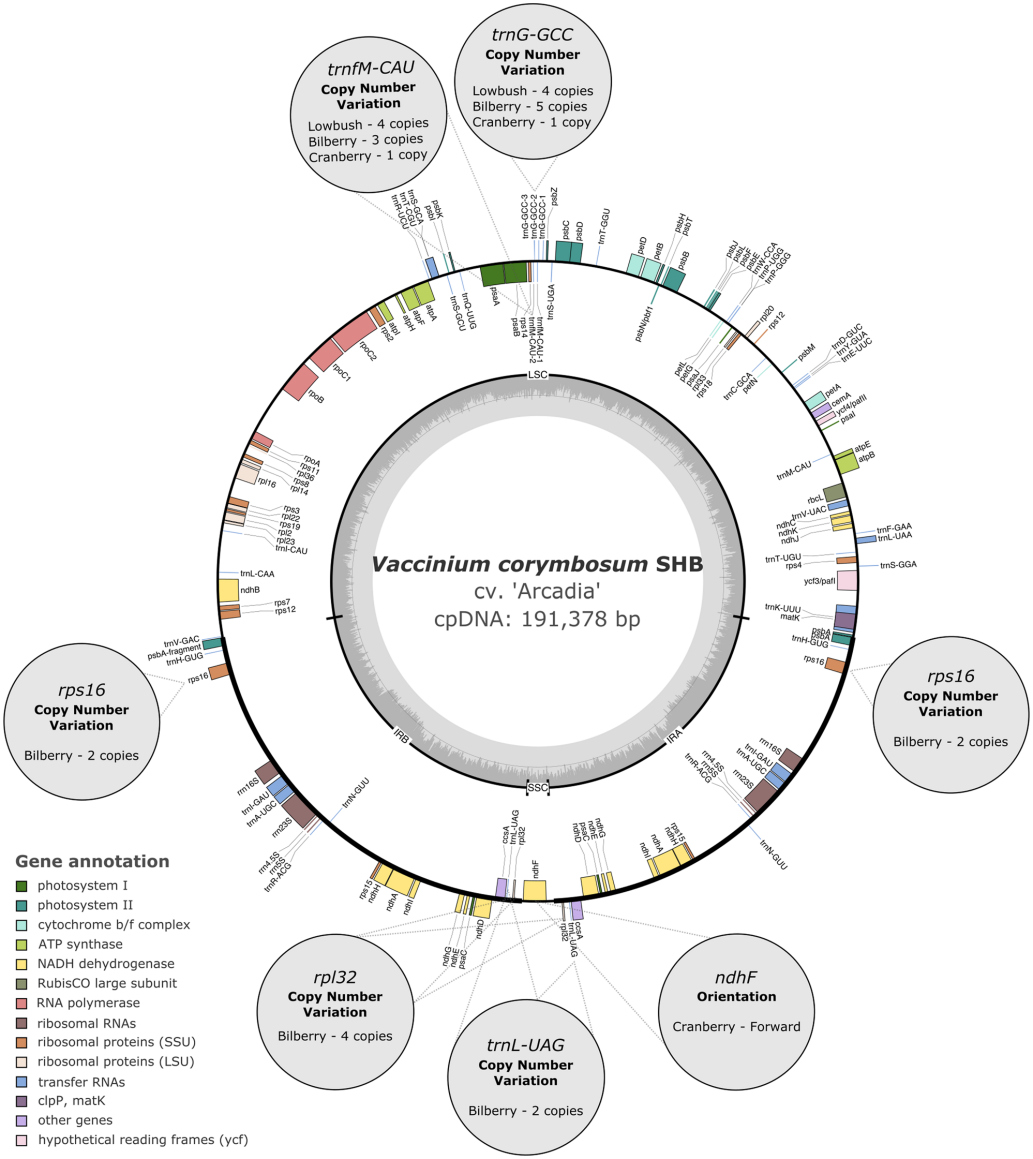
Circular chloroplast genome map of southern highbush blueberry cv. ‘Arcadia’ (*V. corymbosum* hybrids). Outer gray bubbles indicate the variable annotation features among the six *Vaccinium* assemblies. Genes drawn outside and inside the map represent genes transcribed counterclockwise and clockwise, respectively, and the different colors represent their functional annotation. The large single copy (LSC), inverted repeats (IRA and IRB), and small single copy (SSC) regions are shown in the black inner circle. The gray inner circle shows GC content.

**Figure 2:**
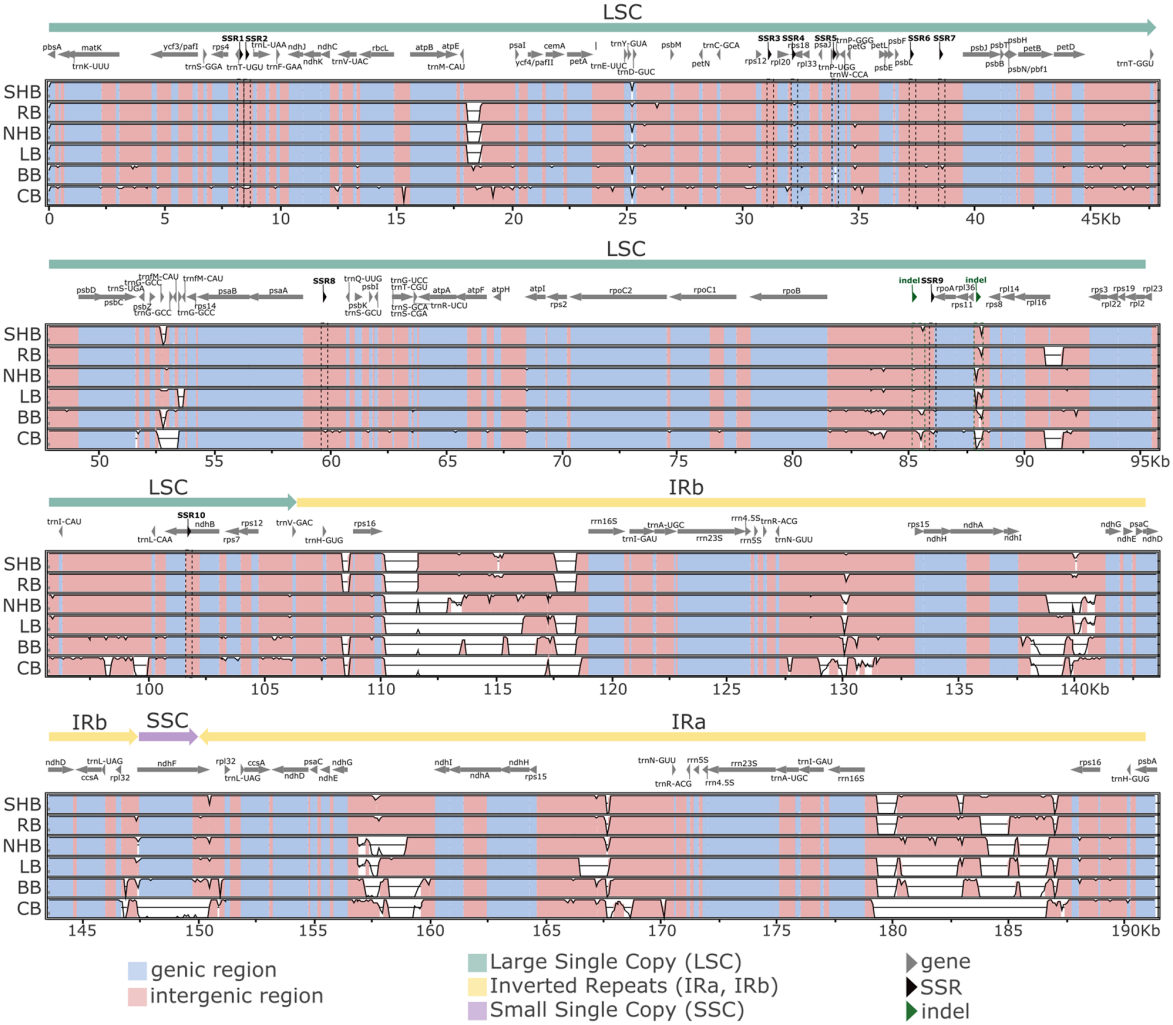
Multiple sequence alignment of *Vaccinium* chloroplast genomes performed with mVISTA. The x-axis represents the coordinates of the southern highbush blueberry chloroplast genome sequence. The y-axis represents the percentage identity ranging from 50 to 100% for each *Vaccinium* species. Divergent regions due to low sequence similarity or the presence of insertions/deletions are shown in white. Polymorphic simple sequence repeat (SSR) and indel regions are highlighted with dashed lines. Abbreviations correspond to the species common names as follows: SHB (southern highbush blueberry), RB (rabbiteye blueberry), NHB (northern highbush blueberry), LB (lowbush blueberry), BB (bilberry), and CB (cranberry).

The final cpDNA sequences obtained showed a quadripartite structure, with a large single copy (LSC) region ranging between 105,715 and 107,608 bp, a pair of inverted repeats (IRA and IRB) ranging from 35,864 to 43,207 bp, and a small single copy (SSC) region ranging from 2998 to 3038 bp (Fig. 1, Table 1, Supplementary Fig. S1). The SSC region was inverted in the cranberry cpDNA compared to the other assemblies (Figs. 1, 2).

### Gene annotation

The five cpDNA sequences assembled here (SHB, NHB, rabbiteye, lowbush, and bilberry) and the cranberry cpDNA assembly downloaded from GenBank (with minor modifications, see Methods) were annotated for genic features, including ribosomal RNAs (rRNAs), transfer RNAs (tRNAs), and protein-coding genes. For all samples, around 40% of the annotated features had to be manually curated by comparison with annotations available for other plant species (Supplementary Table S1).

All chloroplast genomes contained the same number of unique functional genes (112), including 74 protein-coding genes, 34 tRNAs, and 4 rRNAs (Table 1). However, the genomes differed in the number of copies present for the genes *rpl32*, *rps16*, *trnfM-CAU*, *trnG-GCC*, and *trnL-UAG* (Fig. 1). Most of the copy number variation of genes occurred in the draft sequences of lowbush and bilberry. The LSC contained most of the tRNAs (82.4%) and protein-coding genes (85.1%). The IRs contained all four rRNA genes, 11 protein-coding genes and six tRNA genes, which are therefore duplicated in the chloroplast genomes. The SSC contained only one protein-coding gene (*ndhF*), which is in the reverse orientation in the cranberry assembly (Fig. 1).

Nineteen genes contained introns: ten protein-coding genes, and nine tRNA genes. Among those genes, the *rps12* and *psbA* genes had interesting patterns. For the *rps12* gene, the first exon was predicted to be transcribed in the forward direction, while exons 2 and 3 were encoded in the reverse orientation. The *rps12* gene segment containing exon 1 was separated by around 73 kb from the segment containing exons 2 and 3. The *psbA* gene was the only gene spanning the LSC/IR junction, with the starting portion (236 bp) located in the LSC region and the remaining portion (826 bp) located at the end of the IRA. A fragment of the gene was also present in the IRB region, but this partial copy of *psbA* lacked the gene start. The *psbA* gene segments showed the same length in all assemblies except for lowbush, where the gene start located in the LSC was 386 bp long due to an insertion.

In addition to functional genes, eight gene fragments or pseudogenes were reported by the annotation programs in the six *Vaccinium* assemblies: *accD*, *clpP*, *infA*, *psbG*, *ycf1*, *ycf2*, *ycf15*, *ycf68* (Supplementary Table S2). These gene fragments/pseudogenes were removed from the final annotation files.

### Comparative genomic analysis

The sequence similarity between the six cpDNAs was assessed through multiple sequence alignments, which showed that the *Vaccinium* cpDNAs are highly conserved and syntenic, with most of the variation present in non-coding regions (Fig. 2, Supplementary Fig. S2). The main structural differences found were insertions/deletions around the IR borders and the opposite orientation of the SSC in the cranberry cpDNA when compared to the other assemblies. Due to their high synteny, no difference was observed in terms of the genes surrounding the IR/SSC or IR/LSC junctions for the plastomes analyzed here (Supplementary Fig. S3). Overall, the cpDNAs showed a sequence identity to the consensus ranging between 82.98% (cranberry) and 91.50% (rabbiteye). The most conserved regions were the LSC (94.16–97.23% of identity) and the SSC (91.53–98.51% of identity), while the IR was the most divergent region (69.82–87.64% of identity) (Supplementary Table S3).

### Simple sequence repeats and indel analyses

**Figure 3:**
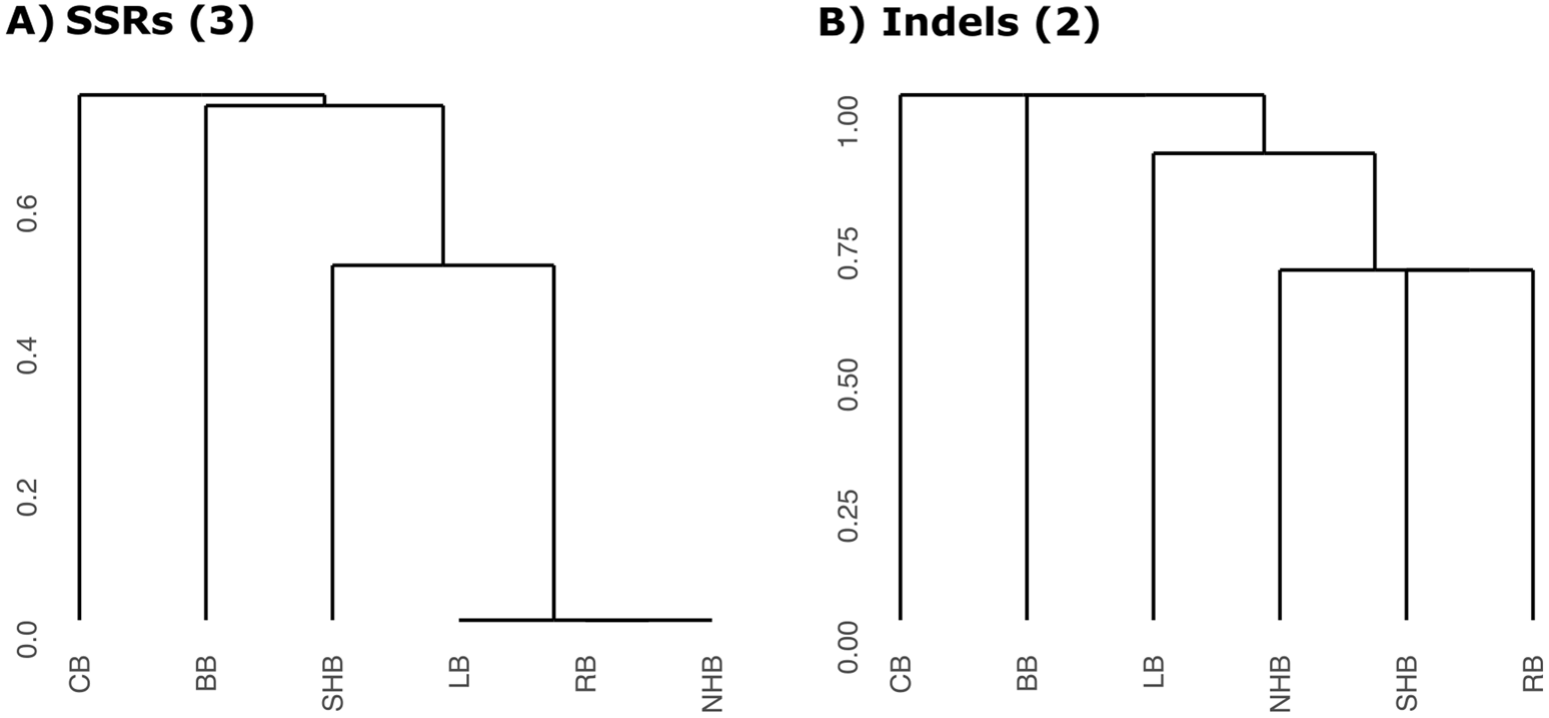
Hierarchical clustering of six economically relevant *Vaccinium* species using different marker types. (**A**) Dendrogram constructed using three simple sequence repeat markers (SSR3, SSR4, and SSR5). (**B**) Dendrogram constructed using two indel-containing regions (“*rpl36*-*rps8”* and “*rpoB*-*rpoA* (3)”). SHB: southern highbush blueberry, NHB: northern highbush blueberry, RB: rabbiteye blueberry, LB: lowbush blueberry, BB: bilberry, CB: cranberry.

The six *Vaccinium* cpDNA assemblies were screened for the presence of simple sequence repeats (SSRs), identifying between 77 (lowbush) and 109 (rabbiteye) SSRs (Supplementary Table S4, Supplementary Table S5). Mononucleotide repeats were the most frequent repeat type and compound repeats were also mainly composed of mononucleotide repeats. In terms of SSR density, the inverted repeats contained ~ 0.75 SSRs/kb, twice as many as the single copy regions (~ 0.34 SSRs/kb). However, SSRs with mononucleotide repeats and located at repetitive regions are less suitable for primer design and genotyping. Therefore, to inspect the usefulness of SSRs as molecular markers, we looked for polymorphisms among the six *Vaccinium* plastomes considering SSRs with di-, tri-, tetra-, penta-, and hexanucleotide repeats located at single copy regions. A total of 10 polymorphic SSR loci were detected (Fig. 2, Supplementary Table S6). Bilberry and cranberry showed greater variation at these loci, while SHB, rabbiteye, NHB and lowbush generally shared the same alleles (Supplementary Fig. S4). The 10 SSRs could only differentiate cranberry, bilberry and SHB from the other species, but they were unable to separate the NHB/lowbush/rabbiteye cluster. A minimum number of three SSRs could be used to differentiate cranberry, bilberry and SHB, since including additional SSRs in the clustering analysis did not improve discrimination power (Fig. 3A).

In search for additional markers that would achieve the complete differentiation of the six commercially relevant species, regions within the LSC showing low homology in the multiple sequence alignment obtained with mVISTA were inspected for insertion-deletion (indel) polymorphisms. One indel located at the intergenic region between genes *rpl36* and *rps8* (marker “*rpl36*-*rps8”* hereafter) showed five different alleles in silico, failing only to differentiate NHB from lowbush (Supplementary Table S7). We also examined eight indel-containing regions detected in previous studies aiming to distinguish *Vaccinium* species^24,32^. Out of those, the marker “*rpoB*-*rpoA* (3)”^24^ was polymorphic and featured different alleles when comparing NHB and lowbush (Supplementary Table S7). Therefore, the combined use of the marker “*rpl36*-*rps8”* identified here and the marker “*rpoB*-*rpoA* (3)” reported previously^24^ allowed for the in silico discrimination between the six commercially relevant *Vaccinium* species analyzed herein (Fig. 3B).

### Phylogenetic tree

The whole plastome alignment of 18 Vaccinioideae species yielded homologous sequence blocks comprising a total of 84,934 bp in length. Most of the sites were conserved across the species, and 8206 single nucleotide polymorphisms (SNPs) were detected. Out of those, 3357 were parsimony-informative and 4849 were singletons (i.e., mutations appearing only once among the sequences).

**Figure 4:**
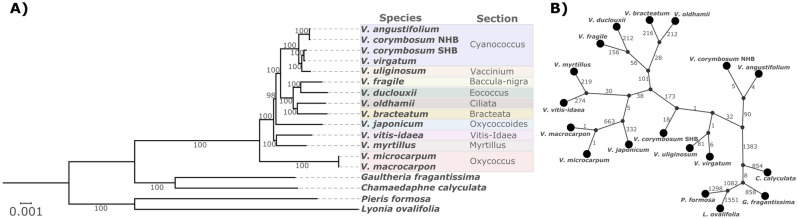
Phylogenetic and haplotype network analyses of whole chloroplast genomes of *Vaccinium* species. (**A**) Maximum likelihood phylogenetic tree. Different shades of colors represent different *Vaccinium* sections. Branch labels indicate the bootstrap support values. The scale bar represents nucleotide substitutions per site. Four taxa from different genera of the *Vaccinioideae* subfamily (*Chamaedaphne calyculata*, *Gaultheria fragantissima*, *Lyonia ovalifolia*, and *Pieris formosa*) were used as outgroups to root the tree. (**B**) Haplotype network showing the mutational steps separating the *s*pecies. Segment length is not proportional to the number of mutations.

A maximum likelihood tree was reconstructed to show the phylogenetic relationships among the species (Fig. 4A). *Vaccinium* species belonging to different sections were supported in the phylogenetic tree, except for the *Cyanococcus* section which was not monophyletic. The species *V. uliginosum* is classified as in the section *Vaccinium*, however it was placed among the species in section *Cyanococcus*. Within the *Cyanococcus* section, it is also noteworthy that the SHB cpDNA was more closely related to *V. virgatum* (rabbiteye) than to *V. corymbosum* (NHB), while NHB showed a closer relationship to *V. angustifolium* (lowbush). The same topology was obtained when reconstructing a majority-rule consensus Bayesian inference tree based on the whole chloroplast alignment (Supplementary Fig. S5) and when reconstructing the ML tree from concatenated protein-coding genes (Supplementary Fig. S6).

Despite considering a large chloroplast genomic region, only few mutational steps separated haplotypes of closely related species based on the whole chloroplast alignment. For example, 26 polymorphisms differentiated SHB from rabbiteye, nine separated NHB from lowbush, and two differentiated cranberry from its wild relative *V. microcarpum* (Fig. 4B). Some allelic variants at the tips of the network were species-specific and could also serve as potential SNP markers for berry crop authentication.

## Discussion

Since the first *Vaccinium* chloroplast DNA sequence was published in 2013^22^, next-generation sequencing technologies have enabled the assembly of plastomes for additional species in this genus, making ten *Vaccinium* cpDNAs available to date^22–29^. Here, we performed de novo assembly of the plastomes of five additional *Vaccinium* samples, focusing specifically on crops of economic importance (four cultivated blueberry types and bilberry).

The highest quality complete plastome assemblies were obtained for SHB and rabbiteye, which were sequenced using long reads from the PacBio platform. The availability of long reads allowed the assembly of the entire cpDNA as a single contig, similar to the assembly done for cranberry using the same technology^23^. Although the remaining species were sequenced with Illumina short reads and the assemblies were split into more than one contig, the use of a reference cpDNA to order the contigs was able to generate draft plastomes for NHB, lowbush and bilberry containing only a few gaps in their sequences. Despite the clear advantage of using the PacBio platform compared to Illumina reads for resolving the repetitive regions (IRs) and achieving complete cpDNA assemblies, the higher sequencing costs of the PacBio technology hindered its usage for all samples. So far, only the cpDNA of cranberry (*V. macrocarpon*) and its wild relative (*V. microcarpum*) had been completely assembled using long reads^23^. All other published *Vaccinium* plastomes are draft Illumina assemblies.

All six *Vaccinium* cpDNA sequences compared here (five new assemblies and the cranberry plastome^23^) showed the typical circular quadripartite structure for angiosperms, including the two copies of inverted repeats separating the large and the small single copy regions^35^. The length of the *Vaccinium* cpDNA assemblies was also within the range reported for angiosperms (107–218 kb)^21^. However, a drastic reduction in the SSC region was observed among the *Vaccinium* assemblies (~ 3 kb) compared to most plant species (16–27 kb), which has also been reported for other members of the *Ericaceae* family^36–38^.

Most angiosperm chloroplast genomes contain 110–130 distinct genes, approximately 80 genes coding for proteins and other genes coding for 4 rRNAs and 30 tRNAs. For the six *Vaccinium* species analyzed in this study, a total of 112 distinct genes were annotated (74 protein-coding, 34 tRNA and 4 rRNA genes). A recent study comparing the plastomes of five other *Vaccinium* species showed differences in gene content among them^24^. This differs from our findings, where even the most distant taxon in the phylogenetic tree (cranberry) carries the same genes as the other *Vaccinium* species sequenced herein. This discrepancy is likely due to software mispredictions. In our study, we observed that using more than one gene prediction software and performing manual curation were important steps for the proper identification of genes and annotation of introns in chloroplast genomes. For example, in our work, we identified four tRNA genes (*trnfM-CAU*, *trnG-GCC*, *trnS-CGA*, *trnS-GCA*) not previously reported in the cranberry plastome, and five functional genes (*atpF*, *ccsA*, *ndhG*, *ndhK*, *rps16*) that were previously considered pseudogenes for either missing the gene start or containing premature stop codons^22,23^. The accuracy of the gene prediction can impact comparative analyses of gene gain/loss among lineages and may prevent informative sites to be included in phylogenetic analyses.

Instead of a difference in the absolute number of distinct genes, we found copy number variation for five genes. However, these copy number variations warrant further validation, since most of them were identified in the lower-quality assemblies of lowbush and bilberry. Eight gene fragments or putative pseudogenes were identified here, including the *accD*, *clpP* and *infA* genes, which have been previously reported as pseudogenes in cranberry but as functional in other members of the *Ericaceae* family^37^.

Besides the gene content, comparative genomics analyses among the six *Vaccinium* species also revealed high similarity in terms of sequence identity and synteny. One synteny difference identified was the opposite orientation of the SSC in cranberry when compared to the other assemblies. However, it has been shown in other plant species that both SSC orientations can be present simultaneously within the same individual due to chloroplast heteroplasmy^39,40^. Therefore, at this point, we cannot consider the SSC orientation a consistent rearrangement in cranberry. Another structural difference was found in a non-coding region close to the IR/LSC boundaries, where the cranberry cpDNA (shortest plastome) shows a missing fragment of approximately 8 kb when compared to rabbiteye (longest plastome). This difference between assemblies is reflected in the lower sequence identity shown within the IRs. Greater sequence divergence within the IRs was also reported in a previous comparison between five other *Vaccinium* species^24^. Indeed, expansion/contraction of the IRs is one of the major causes for plastome size differences between plant species^35^.

Comparison of the abundance of different SSR repeat units showed that mononucleotide repeats were the most frequent repeat type. Mononucleotide repeats are the most abundant and variable class in other plant species^41,42^; however, their use as molecular markers has been limited given their lower reliability and difficulty to genotype^43^. Simple sequence repeats at repetitive regions also make it difficult to design primers specific to the flanking region. Therefore, we searched for variability only at orthologous SSRs with longer repeat units and located at single copy regions, identifying 10 polymorphic SSRs among the six commercially important *Vaccinium* berry crops. However, these SSRs were unable to distinguish closely related species in the *Cyanococcus* section. In contrast, the combined use of two indel markers (“*rpl36*-*rps8”* and “*rpoB*-*rpoA* (3)”) achieved the complete discrimination of the six species. Additionally, species-specific SNPs were detected throughout the whole cpDNA alignment. Both indels and SNPs could serve as potential molecular markers, especially for berry food product authentication, as we included the major economically important *Vaccinium* species in the analyses. The suitability of these markers for fingerprinting remains to be confirmed after primer development and testing at the laboratory.

Phylogenetic analyses were able to distinguish the species of the genus *Vaccinium* and most of the sections were monophyletic considering the few taxa sampled. An exception was the placement of *V. uliginosum* from section *Vaccinium* among species in the section *Cyanococcus*. Intersectional crosses have generally proved difficult to perform, yielding mostly sterile hybrids^44^. However, successful crosses between *V. uliginosum* and *V. corymbosum* were reported to produce meiotically regular and fruitful hybrids^45^, which reinforce their closer phylogenetic proximity.

Plastome-based phylogenetic relationships in the *Vaccinium* genus are complex, particularly due to the history of hybridization events that could have led to chloroplast capture. In addition, the relationships should be interpreted with caution when analyzing such limited number of taxa and domesticated genotypes. Discordances between the chloroplast phylogeny and previous nuclear phylogenies can be pointed out for cultivated blueberries. The chloroplast genomes of the NHB and SHB genotypes used herein have different origins, with NHB being more closely related to lowbush, and SHB to rabbiteye. In the phylogenetic trees derived from nuclear genome-wide SNPs^46,47^ and SSRs^48^, SHB and NHB genotypes were intertwined and more closely related to each other than to lowbush or rabbiteye. Given the primary contribution of *V. corymbosum* to the genetic background of both NHB and SHB^49^, it is expected that the nuclear genome would reflect the described pattern. On the other hand, the cpDNA will only trace back the maternal line inheritance.

In this study, where the plastomes from different blueberry cultivar accessions were analyzed, artificial hybridizations for breeding purposes are the main hypothesis for the multiple origins of the *V. corymbosum* chloroplast. As interspecific hybridizations have been extensively used in blueberry breeding programs, lowbush and rabbiteye lineages are present in the NHB and SHB genetic background as secondary gene pools. *V. angustifolium* has been used since the beginning of highbush blueberry domestication^50^, with genotypes such as ‘Russell’ and ‘North Sedgewick’ being widely used in crosses. During the development of SHB, several rabbiteye genotypes were used as parents to reduce the chilling requirement of NHB^11,51,52^. Therefore, different cultivars of NHB and SHB might show different plastome clustering patterns based on the maternal pedigree as has been found in rice for example^53^. Expanding this study to additional wild *Vaccinium* species, including wild *V. corymbosum* with different ploidy levels, and multiple individuals would help to clarify their hybridization history and avoid unclear clustering of single accessions^54^.

To our knowledge, this is the first study to report the plastome assemblies for commercially important blueberry and bilberry species. These genomic resources will be valuable for *Vaccinium* breeding programs and for biotechnology and food industries. Given the limitations of taxa sampling of currently available *Vaccinium* plastomes, our results contribute for making more genomic resources available for phylogenetic studies of the genus. By tracing the maternal history through cpDNAs, we reveal insights into the domestication of blueberry crop species. The implication of different maternal chloroplast genomes on plant traits and performance is another area that warrants further investigation.

## Conclusions

In this study, the chloroplast genomes of five economically important *Vaccinium* species were assembled: northern highbush blueberry, southern highbush blueberry, rabbiteye blueberry, lowbush blueberry, and bilberry. We also performed manual curation of gene annotations and comparative analyses of these genomes, including the previously available cranberry plastome sequence. The *Vaccinium* chloroplast genomes were highly conserved in terms of structure and sequence, with some variability found mostly in non-coding regions and at the IR/LSC boundaries. Copy number variation of genes requires further investigation as they could be a result of assembly artifacts in draft genomes. The in silico discrimination between the six *Vaccinium* crops could be achieved using two indel-containing regions and/or species-specific SNPs identified here. The phylogenetic tree based on whole cpDNA alignment showed the presence of distinct maternal genomes in highbush blueberries, highlighting the independent evolution of cytoplasmic and nuclear genomes. In addition, chloroplast phylogenetic analyses did not support the monophyly of the *Cyanococcus* section. The availability of more chloroplast genomes from *Vaccinium* species will provide a valuable resource for future comparative studies and phylogenetic resolution of the genus, and for reconstructing the domestication history of cultivated berry crops.

## Methods

### Plant material

The plant material used in this study to generate the DNA sequences for the assembly of the cpDNAs included the following genotypes: *V. corymbosum* hybrid cv. ‘Arcadia’ (Southern Highbush Blueberry—SHB), *V. virgatum* cv. ‘Ochlockonee’ (Rabbiteye Blueberry—RB), and *V. angustifolium* cv. ‘Brunswick’ (Lowbush Blueberry—LB), *V. myrtillus* genotype ‘OU-L2’ (Bilberry -BB). The SHB, RB, and LB cultivars were obtained from commercial nurseries and maintained at the University of Florida, FL, USA. The bilberry plant material was collected from the coniferous forest in the municipality of Oulu, Finland (64° 59′ 08.1″ N 25° 54′ 12.0″ E) and maintained at the University of Oulu. No special permission was required for sampling the bilberry individual at this location according to the Criminal Code of Finland, Chapter 28, Section 14 (public rights). All the plant materials used in this study were in compliance with relevant institutional, national, and international guidelines and legislation.

For *V. corymbosum* cv. ‘Draper’ (Northern Highbush Blueberry—NHB), whole genome sequence data was already available^34^. Therefore, for NHB, raw Illumina sequences were downloaded from NCBI BioProject (PRJNA494180) and used in the assembly pipeline of this study. For *V. macrocarpon* cv. ‘Stevens’ (Cranberry—CB), the chloroplast DNA assembly was already available^23^. The sequence was downloaded from GenBank (MK715447.1) and used in the downstream comparative analyses.

### DNA extraction and sequencing

The high molecular weight DNA extraction from young leaf tissue and the PacBio long read sequencing for the SHB and rabbiteye samples were carried out at the Arizona Genomics Institute, University of Arizona (Tucson, AZ, USA). Briefly, the high molecular weight DNA was extracted using a modified CTAB method and sheared to mode size of approximately 40 kb using G-Tube. PacBio sequencing libraries were constructed using the Express v2 kit (Pacific Biosciences). Template molecules were size selected on BluePippin for either 35 kb and larger (U1) or 20 kb and larger (S1) methods (Sage Sciences). Sequencing was performed on PacBio Sequel II, in CLR mode with a loading concentration of 50 pmol or larger. PacBio consumables used were PacBio SeqII 1.0 chemistry, 8Mv1 cells and 15 h run time.

Short-read Illumina whole genome sequencing was obtained for the SHB, rabbiteye, lowbush and bilberry samples by extracting genomic DNA from leaf tissue using the CTAB method. DNA library preparation and sequencing were carried out at GENEWIZ LCC. (South Plainfield, NJ, USA). Paired end libraries (2 × 150 bp) were sequenced on an Illumina HiSeq4000 instrument. For bilberry, Illumina paired end library preparation and sequencing were conducted at Sequentia Biotech SL (Barcelona, Spain), using a NovaSeq 6000 instrument (2 × 150 bp). The mean insert size for SHB, rabbiteye, lowbush and bilberry was 325 bp, while the Illumina sequencing data downloaded for NHB^34^ included libraries with five different insert sizes: 470 bp, 800 bp, 4000 bp, 7000 bp and 10,000 bp.

### Long-read assembly and polishing

To select only reads matching *Vaccinium* cpDNA out of the total reads obtained in one SMRT cell, PacBio long reads from SHB and rabbiteye were aligned to the reference cranberry cpDNA sequence using BLASR v.20130815 with parameters “–placeGapConsistently, –hitPolicy randombest, –bestn 1, –minMatch 15, and –minAlnLength 500”^55^. The cranberry plastome was used as a reference because its cpDNA has been completely assembled using long reads. The aligned sequences were converted into FASTQ format using the function “bamtofastq” from the bedtools v2.29.2 software^56^. The retrieved reads were de-novo assembled with Canu v1.9 using the parameters “minReadLength = 1000, minOverlapLength = 500, genomeSize = 200 k, correctedErrorRate = 0.030, and corOutCoverage = 40”^57^. The longest contig generated by Canu was circularized using Circlator v.1.5.5^58^ and polished with the Arrow algorithm implemented in the GCpp v1.9.0 software^59^. Five total rounds of polishing with Arrow were performed for SHB and rabbiteye before moving to a second polishing method. The second polishing step was performed with the software Pilon v1.22^60^ using Illumina short reads and default parameters until no more changes were introduced into the sequence (for up to five successive rounds).

### Short-read assembly and polishing

For NHB and lowbush samples, short Illumina read de-novo assemblies were performed with the NovoPlasty v3.8.3 software with default parameters^61^. The resulting scaffolds were aligned to the SHB cpDNA assembly obtained previously with long-read data, using the “nucmer” tool available in Mummer v4.0^62^. The pairwise alignments were visualized using the Mummer tools “show-coords” and “mummerplot” and the individual NovoPlasty scaffolds were ordered and merged into one pseudo-molecule for each sample according to their placement along the reference SHB cpDNA assembly. A stretch of Ns was inserted at the junction sites between concatenated scaffolds. The SHB assembly was chosen as the reference for scaffolding because it was one of the complete plastome assemblies obtained herein from long reads and expected to be more similar to NHB and lowbush than the complete cranberry plastome assembly previously available.

A similar strategy was used for the bilberry assembly but using a different reference genome for scaffolding as the SHB cpDNA was not finished at the time this assembly was generated. Raw short Illumina reads were aligned to the cpDNA sequence of *Vaccinium oldhamii* (GenBank accession: NC_042713.1)^25^. The mapped reads were then extracted, and de-novo assembled with Spades 3.15.3^63^ and with CAP3 v.20120705^64^. The two assemblies were then aligned to the reference *V. oldhamii* genome, the scaffolds were ordered and then merged into one pseudo-molecule.

The NHB, lowbush, and bilberry assemblies were polished using Pilon v1.22 as described above. The NHB and lowbush assemblies were polished multiple times, until no further changes were introduced into the sequences (i.e., four and three rounds, respectively). The bilberry assembly was subjected to only one round of polishing, because additional rounds inserted sequences into multiple sites, generating tandem repeats.

To obtain a more continuous sequence in the inverted repeat (IR) regions, for each species the sequences of its IRA and IRB were aligned, and the consensus sequence was inserted back into the cpDNA assembly to replace the original IR sequences. Finally, when comparing the IR sequence length in the cranberry assembly downloaded from GenBank, we noticed that two bases were absent from one of the IRs. These nucleotides were inserted into the IR where they were missing, resulting in both IRs having the same length and sequence in the cranberry cpDNA.

### Gene annotation

The cpDNA sequences were annotated to predict gene content and position. Two online tools were employed: (i) GeSeq v2.03 by setting parameters “protein search identity = 70; rRNA, tRNA, DNA search identity = 85; and selecting the 3^rd^ party tRNA annotators ARAGORN v1.2.38 and tRNAscan-SE v2.0.5”^65^; and (ii) CpGAVAS with default parameters^66^. The annotations obtained with the different methods within each tool were not consistent for many genes. Discordant annotations were manually curated as follows: (i) pre-selection of the most frequently predicted coordinates for the annotated feature; (ii) comparison of start and end sequences (~ 10 bp) of tRNA, rRNA, protein-coding genes and exons of intron-containing genes with gene models available for other species in the CpGDB database^67^, including *V. macrocarpon*, *V. oldhamii*, *Arabidopsis thaliana*, *Brassica napus*, *Amborella trichopoda*, and *Populus trichocarpa*; (iii) confirmation of proper start and stop codons for protein-coding genes; and (iv) manual search of genes predicted in only a subset of the species to confirm their absence in the remaining species. Manual curation of gene features was performed for the five *Vaccinium* cpDNAs assembled in this study and for the cranberry plastome.

### Comparative analyses

To investigate the genome structure of the cpDNAs, circular maps were drawn using OGDRAW v1.3.1^68^. The cpDNA assemblies were compared by conducting multiple sequence alignments using mVISTA with the LAGAN mode^69^ and with the EMMA tool in the EMBOSS v6.5.7 software^70^ using the ClustalW v.2.1 aligner^71^. The online tool Multiple Sequence Alignment Viewer v1.21.0^72^ was used to visualize alignments generated with EMMA and to estimate the percentage of identity between sequences. IRScope^73^ was used to investigate IR expansion/contraction and junctions between IRs and single copy regions. Prior to conducting these multiple sequence alignments, the cpDNA sequences were modified to break their circular DNA molecules at the same site as the cranberry cpDNA to ensure that the alignments would start at the same position.

### SSRs and indel detection

Considering the potential importance of SSRs in generating genomic diversity, the cpDNA assemblies were annotated using the MISA-web v2.1 software^74^. The minimum number of repetitions was set at ten for mononucleotide repeats, five for dinucleotide repeats, four for trinucleotide repeats, and three for tetra, penta-, and hexa-nucleotide repeats^22^. Orthologous SSRs were inspected for polymorphisms by visualizing the multiple sequence alignments generated with EMMA in the AliView software^75^. Mononucleotide, compound repeats, and SSRs located at repetitive regions were not considered as they are more difficult to genotype and less useful as molecular markers. The multiple sequence alignments were also used to identify indels in low homology LSC regions according to mVISTA, and to evaluate sequence variation in indel markers reported in previous studies^24,32^. Hierarchical clustering dendrograms were generated with the package ggdendro v0.1.23^76^, considering an Euclidean distance matrix on the basis of the UPGMA (Unweighted Pair Group Method with Arithmetic Mean) clustering method.

### Phylogenetic analysis

To infer the phylogenetic relationships among our sequences and other available chloroplast genomes from *Vaccinium* species, we downloaded the GenBank sequences of *V. oldhami* (NC_042713.1), *V. bracteatum* (LC521967.1), *V. duclouxii* (MK816300.1), *V. fragile* (MK816301.1), *V. uliginosum* (LC521968.1), *V. japonicum* (MW006668.1), *V. microcarpum* (MK715444.1), and *V. vitis-idaea* (LC521969.1). The sequences of other four species from the *Vaccinioideae* subfamily but from different tribes were used as outgroups to root the tree: *Chamaedaphne calyculata* (KJ463365.1), *Gaultheria fragrantissima* (NC_059849.1), *Lyonia ovalifolia* (MW801381.1), and *Pieris formosa* (MW801359.1).

We also downloaded and analyzed the cpDNA of the SHB cv. ‘Sharpblue’ (MZ328079.1)^29^. However, the SHB ‘Sharpblue’ cpDNA showed great dissimilarity from *V. corymbosum* SHB ‘Arcadia’ analyzed herein, raising the hypothesis of potential misidentification of the genotype (Supplementary Fig. S7). To confirm this, we generated whole genome sequencing data of a true-to-type ‘Sharpblue’ cultivar released and maintained by the University of Florida. Hierarchical clustering analysis was performed using SNPs identified in the chloroplast sequence considering short-read Illumina data for the *Vaccinium* crop species generated in this study, the SHB cv. ‘Sharpblue’ (SRA accession: SRR14624419)^29^, and the true-to-type cv. ‘Sharpblue’. For all samples, sequence reads were aligned to the *V. corymbosum* SHB cv. ‘Arcadia’ plastome using BWA v.0.7.8^77^. SNPs were identified with Samtools v1.9^78,79^ and VarScan v2.3.6^80^ following a pipeline adapted for variant calling in chloroplasts^81^. The SNPRelate^82^ package for R^83^ was used to generate a dendrogram based on an identity-by-state dissimilarity matrix using 2644 SNPs. The analyses showed that the true-to-type cv. ‘Sharpblue’ grouped closely with *V. corymbosum* SHB cv. ‘Arcadia’, while the previously published ‘Sharpblue’ diverged from the other SHB samples (Supplementary Fig. S8). Given the unknown genotype and/or species identity of this sample, its cpDNA sequence was not included in the analyses.

First, we used a whole chloroplast genome alignment approach to reconstruct the phylogenetic tree. For this, all assemblies were reordered to start with the *rbcL* gene sequence using Circlator v.1.5.5^58^. We used the HomBlocks pipeline to align the whole cpDNA genomes and determine locally collinear blocks among them^84^. The length of the final concatenated alignment of 18 species was 84,934 bp divided into three blocks. The software IQ-TREE v.2.1.0^85^ and the concatenated alignment were used to automatically estimate the best substitution model (“TVM + F + R4”) based on the Bayesian Information Criterion (BIC) and to reconstruct a maximum likelihood (ML) phylogenetic tree using 10,000 ultrafast bootstraps^86^. The resulting tree was visualized with iTOL v.6^87^.

For comparison with the ML phylogenetic tree, a Bayesian inference (BI) tree was obtained with MrBayes v.3.2.5^88^ starting from the HomBlocks concatenated alignment. The Markov chain Monte Carlo analysis was performed using the parameters “ngen = 700,000, samplefreq = 500, printfreq = 500, diagnfreq = 5000”, with the first 25% of the trees being discarded as burn-in. The consensus tree was visualized with iTOL v.6^87^. In addition, we compared the ML tree obtained with nucleotide sequences of a set of 66 protein-coding genes shared by the chloroplast genomes of the species with available annotations. Sequences were aligned using MAFFT v7.490 with parameters “–maxiterate 1000 –globalpair –adjustdirectionaccurately”^89^. Alignments were further trimmed with Gblocks 0.91b and manually edited where necessary^90^. The aligned nucleotide protein-coding sequences were sorted and concatenated using the AMAS tool^91^. Then, IQ-TREE v.2.1.0^85^ was used to reconstruct a ML phylogenetic tree as mentioned above.

To visualize the mutational steps differentiating the *Vaccinium* species, the HomBlocks alignment was used for haplotype network reconstruction using PopART^92^ with the TCS method^93^.

## Supplementary Information

Supplementary Information 1.

Supplementary Information 2.

## Data Availability

The complete chloroplast genomes and annotations are available at the NCBI database. Accession numbers: *V. corymbosum* hybrid cv. ‘Arcadia’ (SHB): OM791342; *V. virgatum* cv. ‘Ochlockonee’: OM791343; *V. angustifolium* cv. ‘Brunswick’: OM791344; *V. myrtillus* genotype ‘OU-L2’: OM809159; *V. corymbosum* cv. ‘Draper’ (NHB): BK061167. The alignments used for comparative analyses and phylogenetic trees reconstruction have been deposited in the Dryad repository: https://doi.org/10.5061/dryad.08kprr560.

## Abbreviations

SHB: Southern highbush blueberry
NHB: Northern highbush blueberry
RB: Rabbiteye blueberry
LB: Lowbush blueberry
BB: Bilberry
CB: Cranberry
cpDNA: Chloroplast DNA
SSR: Simple sequence repeat
SNP: Single nucleotide polymorphism
ITS: Internal transcribed spacer
IRs: Inverted repeats
LSC: Large single copy
SSC: Small single copy
rRNA: Ribosomal RNA
tRNA: Transfer RNAs
