# Supplementary material for: Chloroplast genome assemblies and comparative analyses of commercially important Vaccinium berry crops
Source: Sci Rep. 2022 Dec 14;12:21600. doi: 10.1038/s41598-022-25434-5 (PMC9751094; doi:10.1038/s41598-022-25434-5)
Supplement: Supplementary file 1 — Supplementary Information 1. [file 41598_2022_25434_MOESM1_ESM.docx]

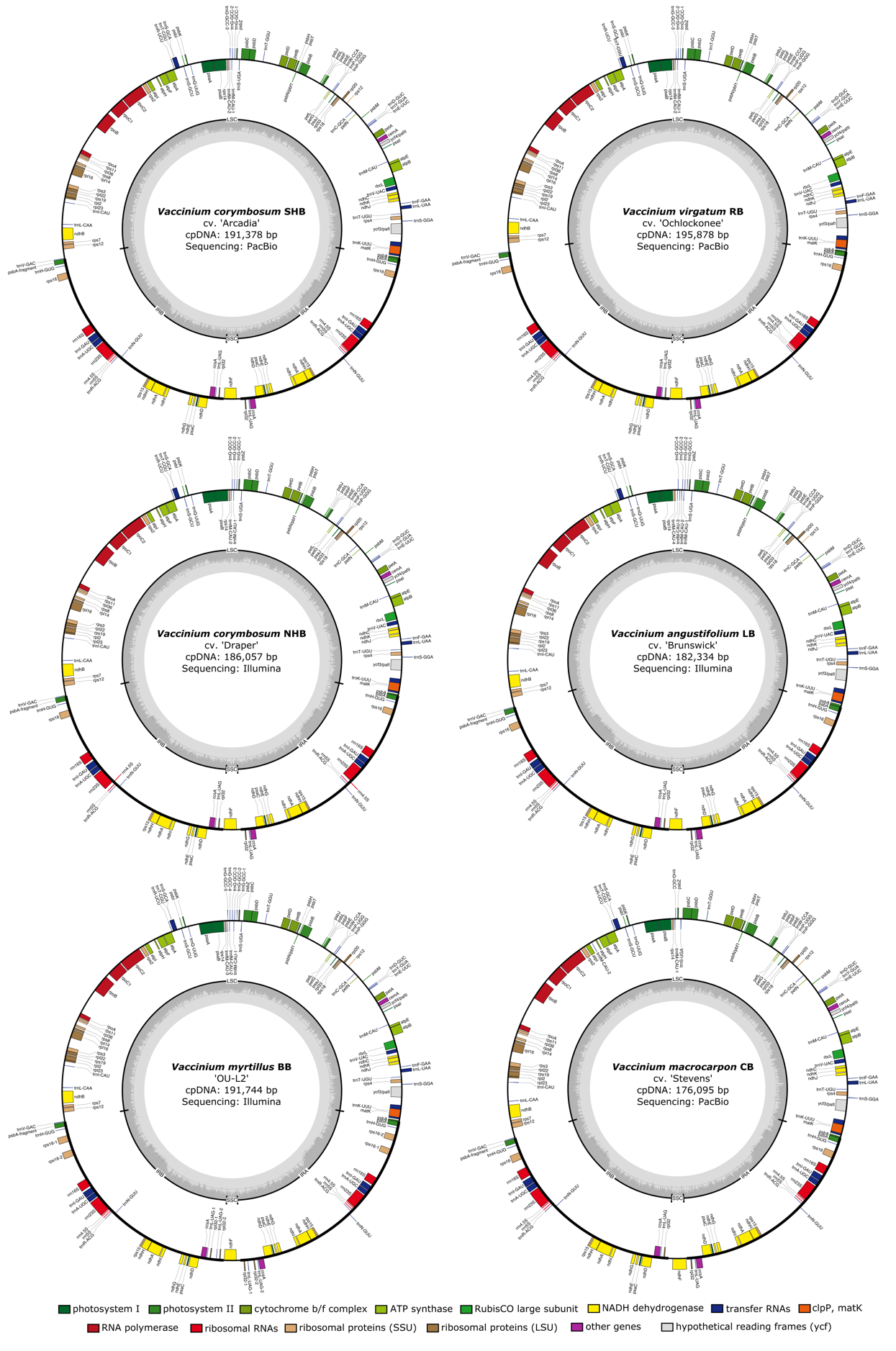
**Additional File 1 - Supplementary Figures**

**Figure S1.** Circular chloroplast genome maps of six *Vaccinium* species. Five species were assembled de-novo and annotated here: southern highbush blueberry (SHB), rabbiteye blueberry (RB), northern highbush blueberry (NHB), lowbush blueberry (LB) and bilberry (BB). Cranberry (CB) was assembled previously (Diaz-Garcia et al., 2019) and reannotated here. Genes drawn outside and inside the maps represent genes transcribed counterclockwise and clockwise, respectively, and the different colors represent their functional annotation. The large single copy (LSC), inverted repeats (IRA and IRB), and small single copy (SSC) regions are shown in the black inner circle. The gray inner circle shows GC content.

**Figure S2.** ClustalW multiple sequence alignment of the complete plastomes of six *Vaccinium* species. SHB: southern highbush blueberry, RB: rabbiteye, NHB: northern highbush blueberry, LB: lowbush blueberry, CB: cranberry, BB: bilberry. ‘Consensus’ refers to the consensus sequence obtained from the alignment.


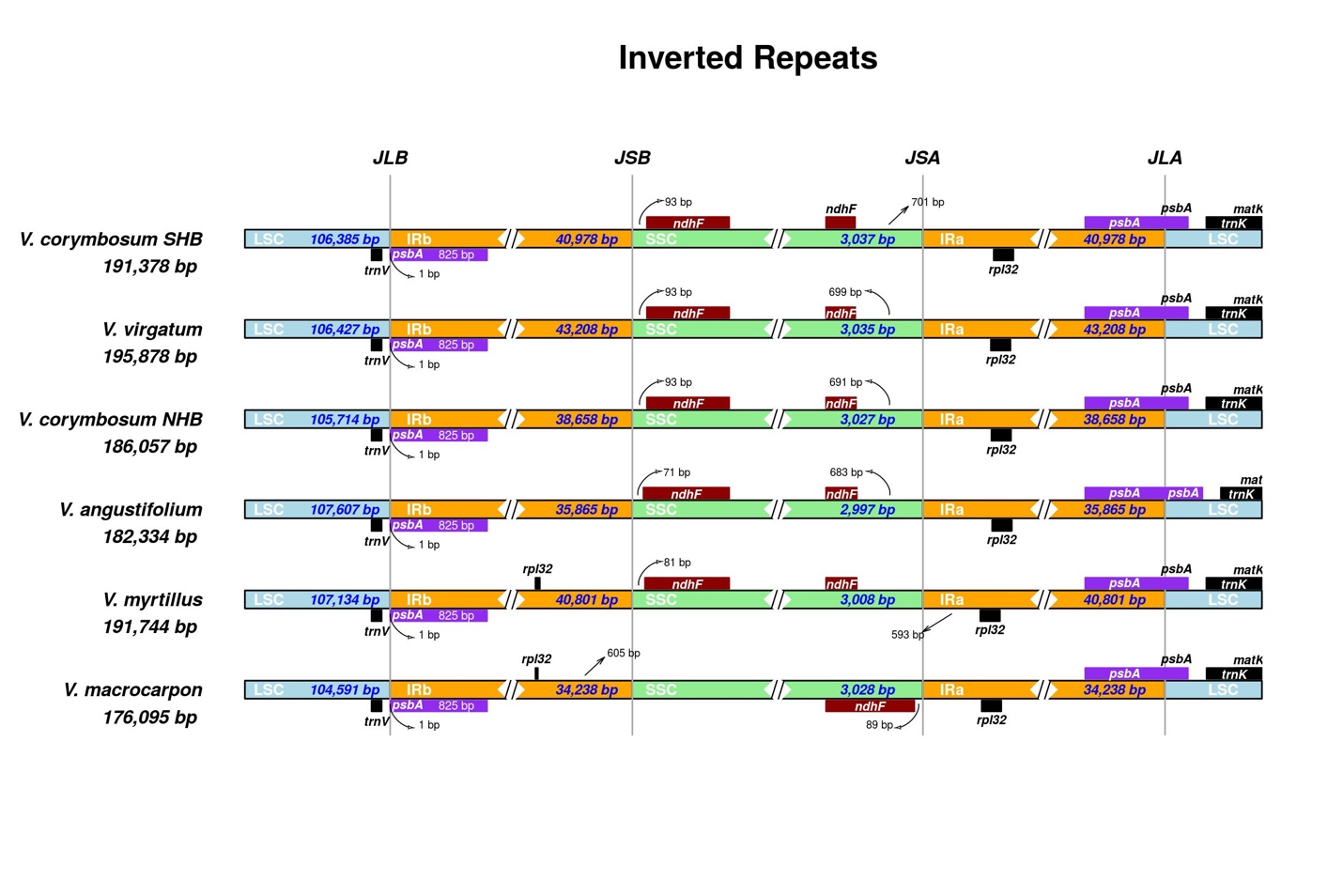


**Figure S3.** Comparison of junction sites between inverted repeats and single-copy regions in six *Vaccinium* species. Genes adjacent to the junction sites are shown as boxes above and below the colored line representing the quadripartite structure of the plastomes (not drawn to scale). In all species compared, the SSC contains only one gene, *ndhF*, which spans most of the SSC sequence (~2240 bp). LSC: large single copy; SSC: small single copy; IRa and IRb: inverted repeats A and B; JLB: LSC/IRb junction; JSB: SSC/IRb junction; JSA: SSC/IRa junction; JLA: LSC/IRa junction; NHB: northern highbush blueberry; SHB: southern highbush blueberry.

**Figure S4.** Sequence variability of ten simple sequence repeats (SSRs) identified in six *Vaccinium* species. For each SSR, the underlined sequence shows the motif identified by the MISA software.

**Figure S4** continues...

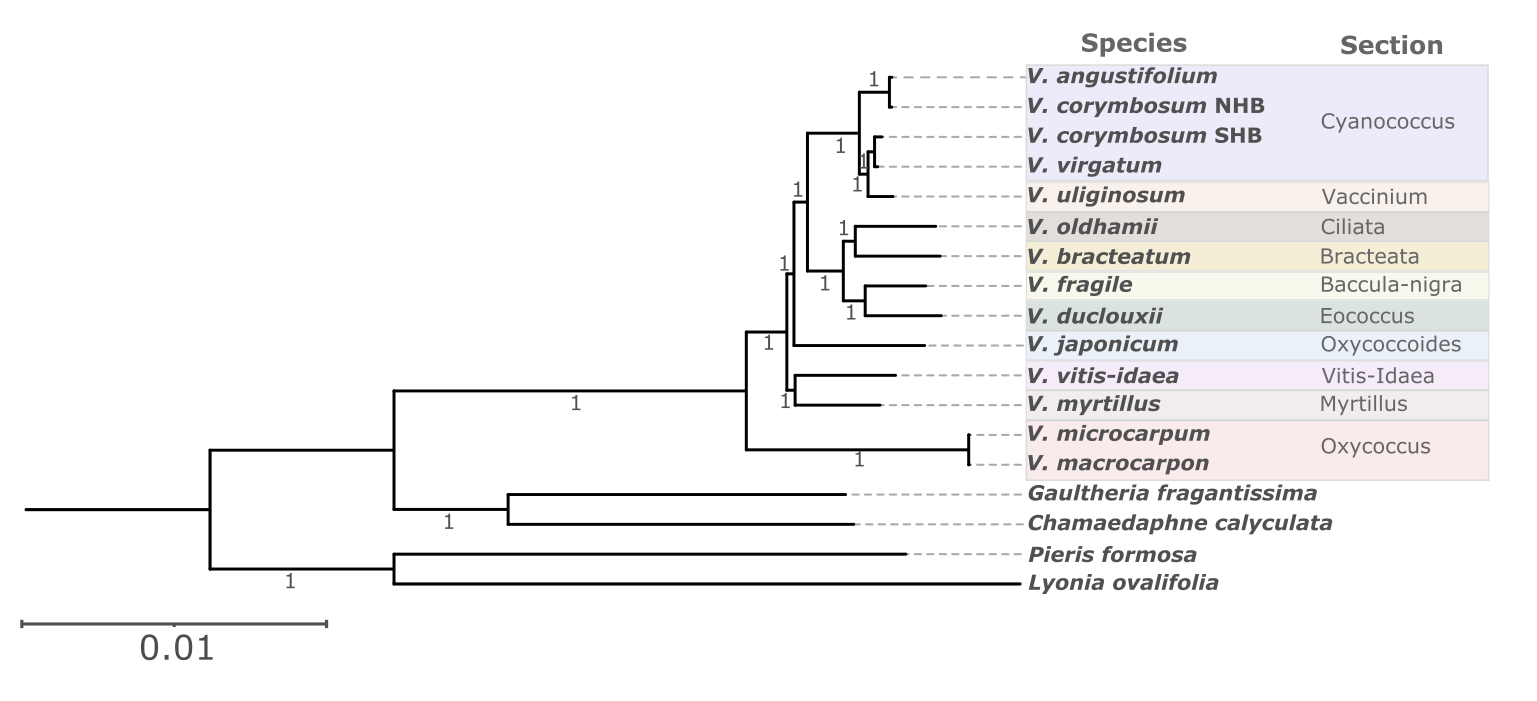


**Figure S5.** Bayesian inference phylogenetic tree from the whole chloroplast genome alignment of *Vaccinium* species. Different shades of colors represent different *Vaccinium* sections. Branch labels indicate the posterior probability values. The scale bar represents nucleotide substitutions per site. Four species (*Chamaedaphne calyculata*, *Gaultheria fragantissima*, *Lyonia ovalifolia*, and *Pieris formosa*) were used as outgroups to root the tree. NHB: northern highbush blueberry; SHB: southern highbush blueberry.


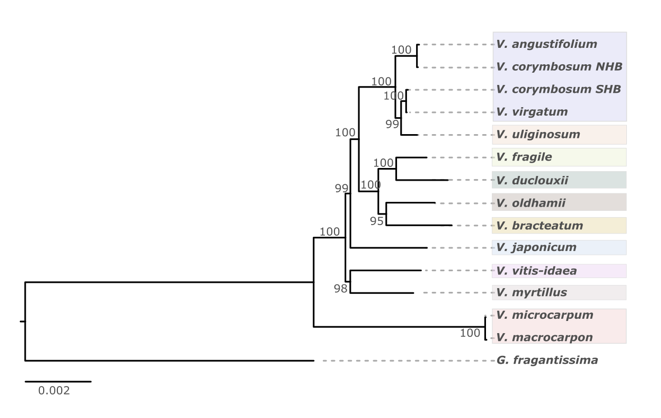


**Figure S6.** Maximum likelihood phylogenetic tree from a concatenated set of 66 protein-coding genes shared by 14 *Vaccinioideae* species. Branch labels indicate the bootstrap support values. The scale bar represents nucleotide substitutions per site. *Gaultheria fragantissima*, a species from a different subgenus of the *Vaccinioideae* family, was used to root the tree. The tree based on protein-coding nucleotide sequences showed the same topology as the tree based on whole chloroplast genome alignment.

**Figure S7.** Bayesian inference phylogenetic tree of whole chloroplast genomes of *Vaccinium* species including the sample reported as cultivar ‘Sharpblue’ by Miao *et al*. (2022). Branch labels indicate the posterior probability values. The scale bar represents nucleotide substitutions per site. Four species (*Chamaedaphne calyculata*, *Gaultheria fragantissima*, *Lyonia ovalifolia*, and *Pieris formosa*) were used as outgroups to root the tree. NHB: northern highbush blueberry; SHB: southern highbush blueberry.

**
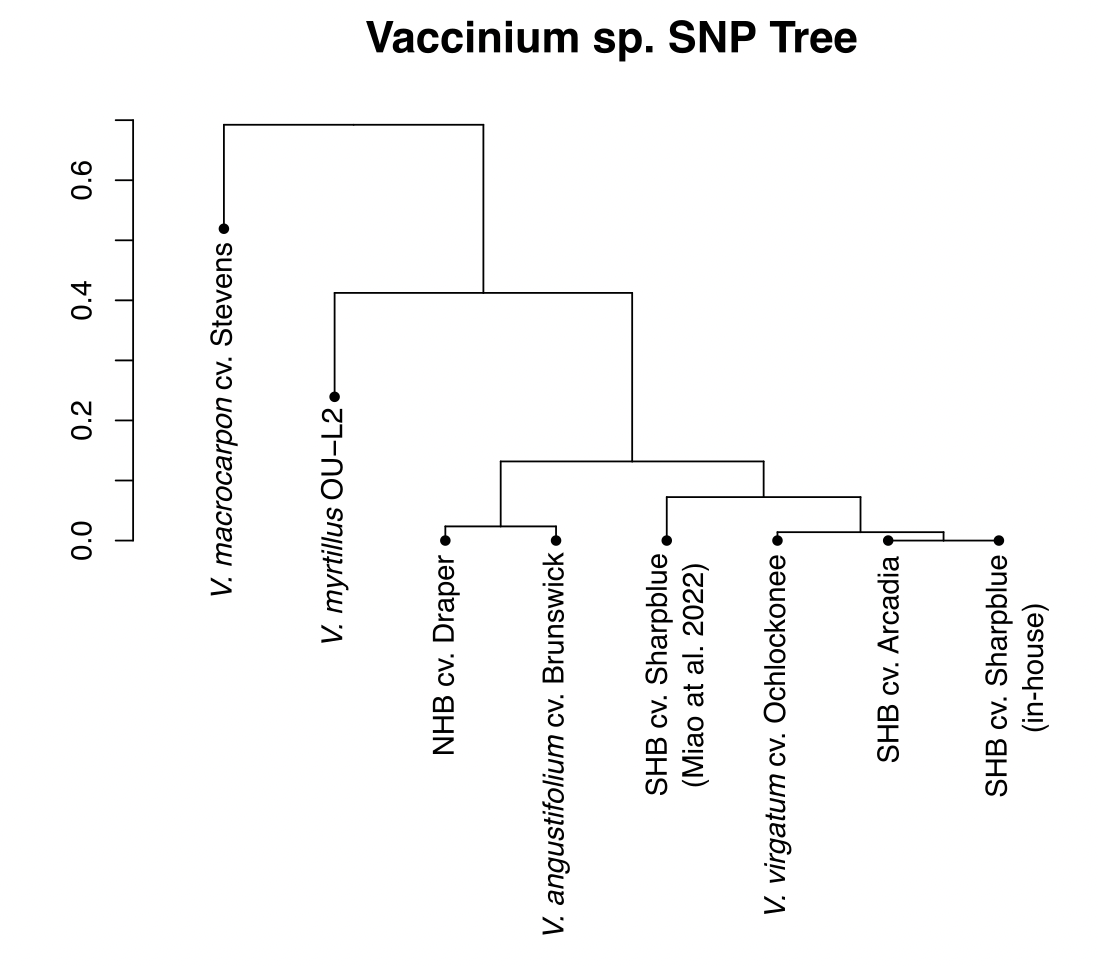
**

**Figure S8.** SNP-based hierarchical clustering of economically relevant *Vaccinium* species including the true-to-type cv. ‘Sharpblue’ and the sample reported as ‘Sharpblue’ by Miao *et al*. (2022). NHB: northern highbush blueberry; SHB: southern highbush blueberry.
